# The Predictive Value of Mutation Screening for Anticipating COVID-19 Waves

**DOI:** 10.3390/pathogens10111464

**Published:** 2021-11-11

**Authors:** Robert Hohan, Petre Milu, Simona Paraschiv, Corina Casangiu, Andreea Tudor, Ovidiu Vlaicu, Leontina Banica, Marius Surleac, Dragos Florea, Dan Otelea

**Affiliations:** 1 “Prof. Dr. Matei Bals” National Institute for Infectious Diseases, 021105 Bucharest, Romania; hohan.robert@gmail.com (R.H.); petre.milu@gmail.com (P.M.); simona.paraschiv@umfcd.ro (S.P.); corina.casangiu@gmail.com (C.C.); vlaicu.ovidiu@yahoo.com (O.V.); leontinabanica@gmail.com (L.B.); marius.surleac@gmail.com (M.S.); dragos.florea@umfcd.ro (D.F.); 2 Faculty of Medicine, “Carol Davila” University of Medicine and Pharmacy, 050474 Bucharest, Romania; 3 Faculty of Biology, University of Bucharest, 077120 Bucharest, Romania; 4 “Prof. Dr. C.C. Iliescu” Emergency Institute for Cardiovascular Diseases, 021105 Bucharest, Romania; tudor.andreea0711@gmail.com; 5 Research Institute of the University of Bucharest (ICUB), University of Bucharest, 060031 Bucharest, Romania

**Keywords:** mutation, screening, prediction, infection, prevalence

## Abstract

Emerging SARS-CoV-2 strains continue to generate difficulties for authorities and health care professionals worldwide due to enhanced transmissibility and/or immune response evasion. The appearance of the Alpha and Delta strains has been associated with substantial increases in the number of COVID-19 cases and associated deaths. Whole Genome Sequencing (WGS) continues to be the gold standard for molecular surveillance of the pandemics but other assays such as mutation genotyping can be used to reduce costs and allocated time. This study investigates the efficiency of mutation screening tests compared to WGS and their predictive value to anticipate future waves. A very high degree of fidelity for this type of assay was found, regardless of the method used. The positive predictive value (PPV) of 4/5 markers was over 95% for the detection of Alpha and Delta variants. By estimating the prevalence of the Alpha and Delta strains using genotyping assays and fitting the data to a mathematical model, a five week period between the point of exponential growth of variant prevalence and a drastic increase in case numbers was found. For that reason, raising awareness about the efficacy of mutation screening could help authorities adopt better measures in the future.

## 1. Introduction

Emerging SARS-CoV-2 (*Realm* Riboviria, *Kingdom* Orthornavirae, *Phylum* Pisuviricota, *Class* Pisoniviricetes, *Order* Nidovirales, *Family* Coronaviridae, *Genus* Betacoronavirus, *Subgenus* Sarbecovirus, *Species* Severe acute respiratory syndrome–related coronavirus, *Virus* Severe acute respiratory syndrome coronavirus 2) strains continue to generate difficulties for authorities and health care professionals worldwide due to enhanced transmissibility and/or immune response evasion. SARS-CoV-2 variant B.1.1.7 (Alpha), followed by B.1.617.2 (Delta) became the dominant strains in certain regions and, later, globally in a time span shorter than three months [1,2]. Not accounting for other important factors, such as public health policies, seasonality, international travel and so on, the spread of these variants also seemed to coincide with the generation of two COVID-19 waves of great magnitude, in terms of number of cases leading to strain on health care systems worldwide. Due to this fact, public health care organisations and researchers have cited the emergence and spread of these variants as the main factor leading to the appearance of these two waves of infections [3]. This has also been supported by experimental evidence [4]. In Romania, the spread of these variants has followed a similar trend to the one observed in other countries and has also been accompanied by two corresponding COVID-19 waves of infection [1,2,5].

These observations have underlined the necessity of tracking the spread of Variants of Concern (VOCs) on an international scale [6], since the emergence of such strains could potentially predict future increases in case numbers and COVID-19 related deaths. Due to their position in the viral genome, such mutations were identified by previous studies [4] as potentially having clinical implications as well, also being involved in better escaping the host immune response or some novel therapies such as monoclonal antibodies. Initially, the main method used to monitor circulating strains was Whole Genome Sequencing (WGS) and it continues to be the gold standard for the molecular surveillance of pandemics. However, other laboratory strategies have been considered in order to decrease costs, human resources involved, and to generate results rapidly. The genotyping approach for SARS-CoV-2 was first used successfully when a deletion of two amino acids (Δ69–70), a hallmark for the B.1.1.7 strain, led to a lack of S gene amplification in PCR testing (S gene target failure - SGTF) on certain commercial tests. This led to the use of SGTF as a screening method for identifying the Alpha strain, with most of the identified strains being later confirmed as B.1.1.7 by WGS. Tests specifically designed to detect key mutations in the genome of VOCs were created [7], which allowed the tracking of SARS-CoV-2 variants on a much larger scale. After the first detection of the Alpha variant in Romania which occurred in January 2021, as described by a previous paper [8], a national program for diagnosing COVID-19 cases, VOC screening by PCR genotyping of key mutations and WGS of SARS-CoV-2 was put in place.

In this study, the data obtained mainly through this program was used to describe the dynamics of these two waves of infection and their correlation to the spread of the Alpha and Delta variants in Romania; this was expected to provide insights into how future such waves might develop.

For a more quantitative description of how these mutations change over time a mathematical model based on the work done by Baranyi and Roberts [9] was used. While less powerful than the more complex COVIDSIM2 developed by Sofonea et al. [10] it had enough predictive power to be an epidemiologically useful tool.

## 2. Materials and Methods

### 2.1. Sample Selection

Samples collected by the Public Health District Authority of Bucharest based on COVID-19 specific clinical epidemiological criteria devised by the National Institute of Public Health and sent to the Molecular Genetics Department of the “Matei Bals” National Institute for Infectious Diseases, Bucharest, Romania for diagnosis by PCR testing. For the period analysed in this study (from the 49th week of 2020 until the 38th week of 2021) a total of 21,279 samples were tested for SARS-CoV-2 RNA, of which 8614 were positive. Furthermore, 3170 were selected for genotyping, with a subset of 403 further tested by WGS.

### 2.2. PCR Testing and Mutation Screening

Viral RNA extraction was performed on the KingFisher™ Flex (Thermo Fisher Scientific, Waltham, MA, USA) platform. PCR amplification for diagnostic purposes was done using Allplex™ 2019-nCoV Assay (Seegene, Seoul, Korea) on the CFX96™ (BioRad, Hercules, CA, USA) thermocycler. For mutation screening, the chosen markers were: SGTF and the N501Y substitution for the Alpha VOC, and the 501N, L452R and P681R versions for the Delta VOC. To perform the genotyping of these markers: TaqPath™ COVID-19 Assay (Thermo Fisher Scientific) to detect SGTF, TaqMan™ SNP Genotyping Assay (Thermo Fisher Scientific) to detect 501N/Y, P681R and Allplex™ SARS-CoV-2 Variants I Assay (Seegene) to detect the L452R substitution were used according to the manufacturer’s specification on CFX96™ and QuantStudio7 (Thermo Fisher Scientific).

### 2.3. WGS Methodology

Viral RNA extraction was performed with MagNA Pure LC Total Nucleic Acid Kit (Roche, Basel, Switzerland). DeepChek Whole Genome SARS-CoV-2 Genotyping kit (Advance Biological Laboratory, Strassen, Luxembourg) was used for reverse transcription and PCR in order to generate amplicons covering the whole viral genome. For DNA library preparation, Illumina DNA prep with the enrichment kit (Illumina, San Diego, CA, USA) was used. DNA libraries were sequenced using Illumina^®^ MiSeq^®^ Reagent Kit v3 and MiSeq platform.

The optimised WGS Bioinformatics pipeline used to generate the SARS-CoV-2 complete genome sequences consisted of a double-assembly method approach in which de novo assembly was combined with multiple rounds of reference mapping, as previously described [8]. This was double-checked using dedicated automatic online SARS-CoV-2 analysis pipelines such as EDGE [11].

### 2.4. Mathematical Modelling for VOC Prevalence Dynamics

In order to determine the key parameters of the changing prevalence of each VOC, as described by the genotyping data, a modified version of the Baranyi model [9] was used. It is based on the logistic function [12] which models the population growth of a group by assuming an intrinsic growth rate ($r_{max}$) and a density dependent crowding effect as the population reaches equilibrium. However, the approach used by Baranyi and Roberts includes the added parameter of the lag time, which allows to better fit data with a long period of time before detection of exponential growth. The equation used was the following:

(1) $$P\left(t\right)=P_{max}+ln\left(\frac{e^{r_{max}\cdot t}-1+e^{r_{max}\cdot t_{lag}}}{e^{r_{max}\cdot t}-1+e^{r_{max}\cdot t_{lag}+P_{max}-P_{min}}}\right)$$

where

P(t), prevalence as a function of time;

*t*, time (in this case, measured in weeks);

$P_{max}$, maximum prevalence;

$P_{min}$, initial prevalence;

$r_{max}$, maximum growth rate;

$t_{lag}$, time elapsed until the start of the exponential phase.

The Levenberg-Marquardt nonlinear least-squares algorithm from the *minpack.lm* package [13] in R [14] was used to obtain the model fit. Furthermore, as the Alpha strain exhibits both a growth and an extinction phase, covering the entire timeline meant using the model twice, the second time with the equation reversed.

## 3. Results

### 3.1. Validation of Mutation Screening Assays by Whole-Genome Sequencing

In order to confirm the validity of the markers described in the previous section, WGS was performed for part of the samples detected with each marker. For the Alpha strain, SGTF was the least sensitive marker due to a number of samples without SGTF that actually showed the 69–70 deletion when sequenced. Co-circulation of the B.1.258 lineage at the time in Romania, which also showed the 69–70 deletion, resulted in some false positive identifications. The other marker, the N501Y substitution, was highly sensitive - none of the samples lacking this mutation belonged to the B.1.1.7 lineage. The sensitivity, specificity, PPV, negative predictive value (NPV), and F1-score of these markers can be seen in Table 1.

The markers used to detect the Delta strain showed a similarly high degree of fidelity. The 501N version, which was initially detected as a consequence of screening for Alpha, also served as a very good proxy for the detection of the Delta VOC, having a sensitivity of 100%. The other two markers (the L452R and P681R substitutions) scored 100% efficiency for all the mentioned parameters (Table 2).

### 3.2. The Dynamics of Alpha and Delta VOC Prevalence

The first detection of an Alpha VOC specific marker (SGTF) occurred at the end of 2020 (30th of December). Afterwards, the proportion of SGTF occurrences remained below 30% for another four weeks. Starting with the fourth week of 2021, the proportion of SGTF, and later N501Y, which became the Alpha VOC marker in usage, grew exponentially until week 12 of 2021. At that point, nearly 100% of the positive samples selected for variant screening showed the N501Y substitution at genotyping. For the next 10 weeks, the proportion of positive cases that had the Alpha specific marker stabilised at nearly 100%.

**Figure 1:**
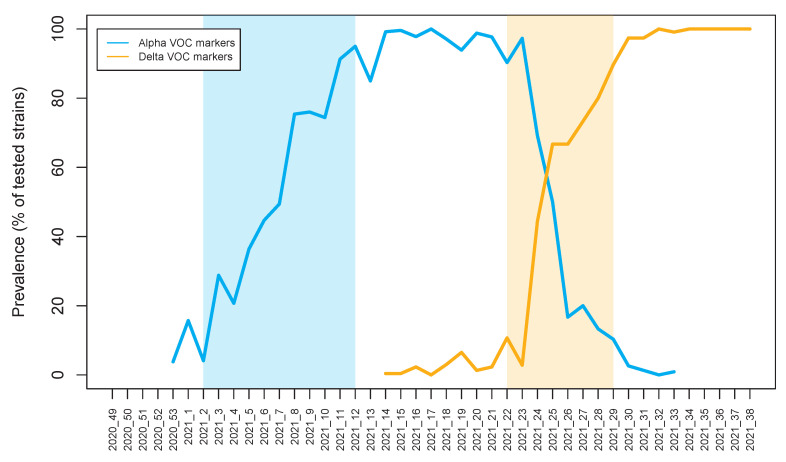
The timeline (expressed in weeks, with each point being formatted as year_week) of the prevalence of the two VOCs under consideration: Alpha (blue) and Delta (orange). The shaded areas represent the period of exponential growth for each variant respectively as calculated from the model fit.

From week 14 until week 23 of 2021, cases that did not show the N501Y substitution (501N version) were sporadic. However, after this point, the number of 501N cases increased rapidly, replacing N501Y variants almost completely in just 4 weeks. Also, after week 25, when testing for the L452R and later P681R Delta specific substitutions was initiated, the increase in proportion of these markers followed an identical trend to the increase of 501N cases. After week 29, the proportion of samples that showed these Delta-specific markers stabilised at nearly 100% (Figure 1).

**Figure 2:**
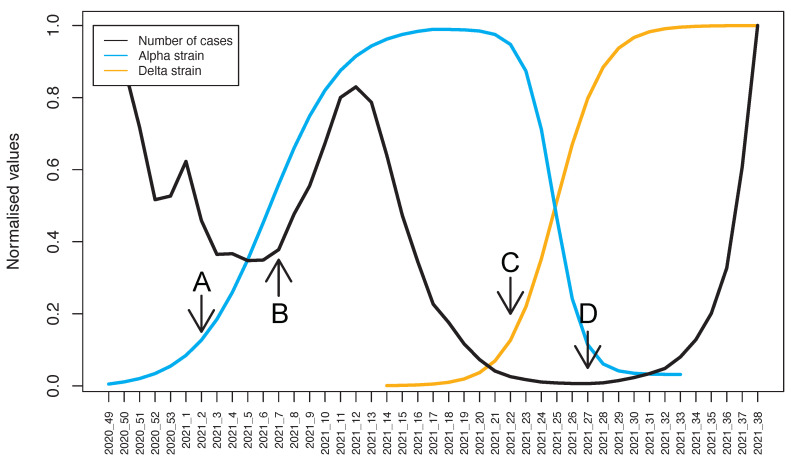
Prevalence data (fitted to the Baranyi model) and number of new infections. To normalise the values for the new weekly infections the number was divided by the maximum number of infections. The four labelled time-points are: A (start of the exponential phase for Alpha VOC), B (beginning of the third COVID-19 wave), C (start of the exponential phase for Delta VOC), and D (beginning of the fourth COVID-19 wave).

Following the implementation of the Baranyi model (Figure 2), the Alpha strain was determined to have had a maximum growth rate ($r_{max}$) of 0.43 (SE = 0.06, *p* < 0.001), having started its exponential phase at week 02/2021 (value obtained by finding the maximum value of the second derivative), and reaching a 90% prevalence by week 12/2021. For the second part of its evolution, the extinction phase of this strain had started by week 23/2021, with a maximum rate of 1.10 (SE = 0.17, *p* < 0.001), having reached a 10% prevalence by week 26/2021. When it comes to the Delta strain, the growth rate was found to be 0.67 (SE = 0.10, *p* < 0.001), with the lag phase ending at week 22/2021, and a 90% prevalence was reached by week 29/2021.

Looking at these parameters, a few differences can be identified. The first one is regarding $r_{max}$, with the Delta strain exhibiting a 55% higher rate of growth. Furthermore, the time it took (since the onset of exponential growth) for each strain to get to 90% prevalence was 10 weeks for Alpha and only 7 weeks for Delta.

## 4. Discussion

For identification of circulating VOCs, the ECDC has urged caution when referring to the usage of solely SGTF or SNP identification assays, advising laboratories to also perform WGS or at least sequencing of the S gene to confirm the results [7]. Nevertheless, this study has found a very high degree of fidelity for this type of assay, regardless of the method that was used. The PPV of four out of the five markers used was over 95% for the detection of Alpha and Delta variants. In other words, mutation screening assays served as a reliable proxy for sequencing in 95% of cases for these tests at that time. While remaining complementary, these methods show important advantages when compared to NGS. The costs in terms of equipment, reagents and personnel are significantly lower and the time required to perform them is much smaller. This makes such assays easily scalable for performing large numbers of tests if subtyping is the end goal, without losing much in terms of accuracy of detection and are thus very valuable at a population level.

Analysing these two waves of infection, a few observations can be made. The third one started from an already high number of cases because the previous wave hadn’t completely subsided, therefore when the Alpha strain started to become the dominant one, cases quickly rebounded. Furthermore, having reached 100% prevalence, the presence of the Alpha VOC did not prevent case numbers from being lowered. This could have been caused by vaccination efforts at the time, seasonality and other public health care measures. The replacement of the Alpha strain by Delta can be clearly observed in Figure 2 when the prevalences of the two variants intersect at a midpoint around week 25.

When developing the mathematical model, multiple approaches were considered from linear models [15] to more complex multi-parameter ones based on a series of differential equations [16]. From these, the one proposed by Baranyi [9] was eventually used, albeit in a closed form, as it both described the changing prevalence well, with the added benefit of the extra parameter of the lag time ($t_{lag}$), which in an epidemiological context is an important element to estimate. More specifically in this study, the accurate determination of the lag time permitted a reliable correlation with the onset of new waves of infection. Furthermore, the implementation of a custom equation permitted in the case of the Alpha strain an evaluation of both the growth and extinction phases, which could be useful in the eventuality of a subsequent variant replacing the Delta strain.

The main value of performing mutation screening assays is perhaps their predictive value. For the third and fourth waves of COVID-19 cases occurring in Romania, the points of inflexion that corresponds to the beginning of the exponential growth phase of each VOC’s prevalence (Alpha and Delta respectively) were situated 5 weeks prior to the beginning of the exponential growth phase of case numbers per week (Figure 2). These genotyping assays yield optimal results when the used markers are frequently reevaluated based on the emerging epidemiological data regarding new strains and subsequently changed in order to facilitate the early detection of new VOCs. The selection of these mutations must be done with care, taking into consideration both a high prevalence in the new variant, but also for it to be sufficiently distinctive for that strain. If mutation screening assays are performed regularly and the sample is representative enough, the rapid increase in prevalence of a new VOC could predict a subsequent increase in case numbers [17]. In general, public health authorities adopt measures to limit the spread of SARS-CoV-2 only after the number of cases increases above a threshold, which raises the following question: can the efficiency of these measures be increased if they are adopted even earlier, when the prevalence of a VOC increases exponentially? For that reason, raising awareness about the efficacy of mutation screening for predicting waves of infection could help authorities adopt better measures in the future.

## 5. Conclusions

This study used a set of methods, both molecular (genotyping) and mathematical, that could serve as complementary tools to whole genome sequencing for a quick and reliable way of tracking the rapid and frequent changes in the mutational landscape of SARS-CoV-2. The main advantages of this approach were not only comparable detection capabilities with a substantially lower cost, but also the ability to identify a 5 week gap between the exponential growth of a new strain and the eventual beginning of a new wave of infections.

## Figures and Tables

**Table 1:** Validation of the two PCR assays used to screen for the Alpha (B.1.1.7) strain with WGS.

Test for Alpha VOC	Target	Sensitivity	Specificity	PPV	NPV	F1-Score
TaqPath (n = 120)	SGTF (del 69/70)	89%	97%	98%	85%	0.93
TaqMan SNP (n = 166)	501Y	100%	92%	95%	100%	0.97

**Table 2:** Validation of the three PCR assays used to screen for the Delta (B.1.617.2) strain with WGS.

Test for Delta VOC	Target	Sensitivity	Specificity	PPV	NPV	F1-Score
TaqMan SNP (n = 166)	501N	100%	95%	89%	100%	0.94
Allplex Variants II (n = 25)	452R	100%	100%	100%	100%	1
TaqMan SNP (n = 38)	681R	100%	100%	100%	100%	1

## Data Availability

The data presented in this study are available in the main text, figures, tables.

## Abbreviations

The following abbreviations are used in this manuscript:

NGS	Next Generation Sequencing
SGTF	S-gene Target Failure
SNP	Single-nucleotide Polymorphism
VOC	Variant of Concern
WGS	Whole-Genome Sequencing
